# MiRNA-548ah, a Potential Molecule Associated with Transition from Immune Tolerance to Immune Activation of Chronic Hepatitis B

**DOI:** 10.3390/ijms150814411

**Published:** 2014-08-19

**Authors:** Tong-Jing Xing, Hong-Tao Xu, Wen-Qing Yu, Bian Wang, Jing Zhang

**Affiliations:** Department of Infectious Diseases, Taizhou People’s Hospital, Taizhou 225300, Jiangsu Province, China; E-Mails: xuhongtaotaizhou@sina.com (H.-T.X.); yuwenqing125@sina.com (W.-Q.Y.); wangbian0942@sohu.com (B.W.); 1987zhangxinxin@163.com (J.Z.)

**Keywords:** chronic hepatitis B, microRNA, immune tolerance, immuneactivation

## Abstract

Objective: The present study aims to identify the differently expressed microRNA (miRNA) molecules and target genes of miRNA in the immune tolerance (IT) and immune activation (IA) stages of chronic hepatitis B (CHB). Methods: miRNA expression profiles of peripheral blood mononuclear cells (PBMCs) at the IT and IA stages of CHB were screened using miRNA microarrays and authenticated using a quantitative real-time polymerase chain reaction (RT-PCR). Gene ontology (GO) and the Kyoto encyclopedia of genes and genomes (KEGG) were used to analyze the significant functions and pathways of possible target genes of miRNAs. Assays of the gain and loss of function of the miRNAs were performed to verify the target genes in THP-1 cell lines. The luciferase reporter test was used on 293T cells as direct targets. Results: Significantly upregulated miR-548 and miR-4804 were observed in the miRNA microarrays and confirmed by RT-PCR in PBMCs at the IT and IA stages of CHB. GO and KEGG analysis revealed that MiR-548 and miR-4804 could be involved in numerous signaling pathways and protein binding activity. IFNγR1 was predicted as a target gene and validated as the direct gene of MiR-548. Significant negative correlation was found between the miR-548ah and mRNA levels of IFN-γR1 in CHB patients. Conclusions: The abnormal expression profiles of miRNA in PBMCs could be closely associated with immune activation of chronic HBV infection. miR-548, by targeting IFN-γR1, may represent a mechanism that can facilitate viral pathogenesis and help determine new therapeutic molecular targets.

## 1. Introduction

The Hepatitis B virus (HBV) infection remains a serious public health problem worldwide despite the wide use of the HBV vaccine. Over 350 million people have been chronically infected with HBV, and approximately 20% to 40% of them will develop liver cirrhosis and liver cancer from chronic episodes of inflammation [1]. Numerous studies suggest that HBV is not directly cytopathogenic for infected hepatocytes. The host immune response, especially the virus-specific T cell response, plays an important role in the viral clearance and pathogenesis of liver diseases [2,3]. Patients suffering from chronic hepatitis B (CHB) have weak and limited T cell responses, whereas those with acute hepatitis have vigorous and polyclonal T cell responses, which can help them completely clear the virus [4]. The lack of virus-specific T cell response in the former case might be attributed to various factors, such as T cell deletion, anergy, exhaustion, ignorance, and T cell dysfunction [5], but the exact mechanism is not yet clear. In addition, impairment of functions of dendritic cells, natural killer cells, and natural killer T cells occur during chronic HBV infection [6,7].

MicroRNA (miRNA) is a small, endogenous, non-coding RNA that modulates gene expression at the post-transcriptional level. MiRNA plays an important function in various activities of organisms and is involved in the development of diseases such as infection and cancer [8,9]. Recent studies have shown that miRNA molecules may have important functions in the development and differentiation of immune cells as well as in the regulation of immune responses [10,11]. Certain miRNA molecules operate in the negative feedback loops of the immune system, whereas other miRNAs help amplify the immune response by repressing the response inhibitors [12,13]. MiRNAs exhibit spatial and temporal specificities in the regulation of the immune system. For example, immune-related miRNA genes serve as “fine-tuners” of gene expression rather than “switchers” [12]. Furthermore, regulation by miRNAs is not antigen specific. Rather, they may target a specific gene locus in a specific cell type and affect its performance in a specific time and space. Studying the hereditary, conditional resting, and activation of miRNA genes might provide a foundation for immunological studies and contribute to development of novel interventions for the immune-related disease [13].

Acquired chronic HBV infection can be divided into four phases according to the characteristics of its immune response: immune tolerance (IT), immune activation (IA), immune control, and immune reaction [14]. The IT phase is characterized by active HBV replication, positivity for HBeAg, and normal alanine aminotransferase (ALT) levels. The IA phase is characterized by decreases in HBV DNA and raised ALT levels. CHB activation may be associated with the enhancement of T helper 1 cells, increases in HBcAg-specific cytotoxic T lymphocytes, and decreases in HBcAg-specific regulatory T cells [15]. Several studies have found that the abnormal expressions of certain miRNA molecules (miR-155, miR-181, *etc.*) are related to the pathogenesis and disease progression of chronic HBV infection [16,17]. However, whether or not exists a molecular mechanism of miRNA in the transition from IT to IA of CHB is unclear. In the present study, miRNA expressions in peripheral blood mononuclear cells (PBMCs) of the IT and IA phases of CHB were screened using miRNA microarrays and authenticated using quantitative real-time polymerase chain reaction (RT-PCR). Target genes of the miRNAs associated with the IA of CHB were then predicted and identified.

**Figure 1:**
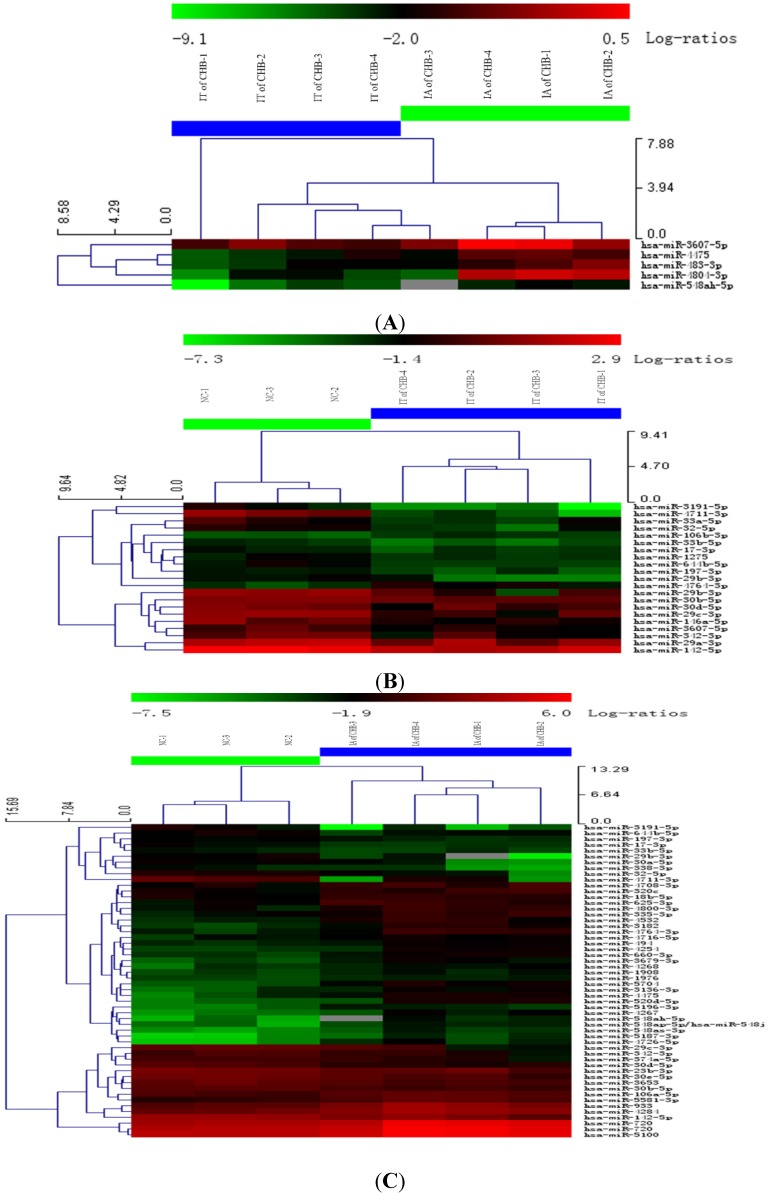
Hierarchical cluster analysis using total miRNA of PBMC in IA and IT stages of Chronic HBV infection. (**A**) IT of CHB *vs*. IA of CHB; (**B**) IT of CHB *vs*. NC; (**C**) IA of CHB *vs*. NC. Legend: IT, immune tolerance; IA, immuneactivation; NC, normal control; CHB, chronic hepatitis B. Note: (1) Red represents that the upregulation of miRNA; Green represents a downregulation of miRNA; (2) The PBMCs of three subjects fromthe samegroup were pooled and four or three pools were analyzed in the microarray groups.

## 2. Results

### 2.1. Different Expressions of miRNA Molecules in PBMC in IT and IA Stages of CHB

miRNA microarray was used to detect the miRNA expressions of PBMCs during the IT and IA stages of CHB. Hierarchical cluster analysis was also performed. Results are shown in Figure 1A–C. Compared with the normal controls, 2 significantly upregulated and 18 significantly downregulated miRNA molecules were identified in PBMCs during the IT phase of CHB; 33 significantly upregulated and 19 significantly downregulated miRNA molecules were also identified in PBMCs during the IA phase of CHB. Compared with the IT phase of CHB, 5 significantly upregulated miRNA molecules (hsa-miR-548ah-5p, hsa-miR-4804-3p, hsa-miR-483-3p, hsa-miR-3607-3p, hsa-miR-44475), were identified in PBMCs during the IA phase of CHB. In particular, the expressions of hsa-miR-548ah-5p and hsa-miR-4804-3p more significantly upregulated in the IA phase of CHB (5.1 and 5.9 times, respectively).

### 2.2. Detection of miRNA Molecules by Quantitative Real-Time PCR

Two miRNA molecules with abnormal expressions (over 5-fold upregulation), including hsa-miR-548ah-5p and hsa-miR-4804-5p, were selected and detected using RT-PCR to authenticate the microarray results. Compared with healthy controls and IT phases of CHB, the expression levels of hsa-miR-548ah-5p and hsa-miR-4804-3p were significantly upregulated in patients with the IA phases of CHB (*F* = 28.9, 79.2, *p* < 0.01) (Table 1).

**Table 1:** Comparison of miR-548ah and miR-4804 expression (Δ*C*_t_, *x* ± *s*).

Groups	*n*	miR-548ah ^Δ^	miR-4804 ^※^
IA of CHB	24	4.6 ± 1.9	9.7 ± 2.6
IT of CHB	23	9.2 ± 2.5	20.8 ± 2.7
HC	24	5.8 ± 2.1	16.5 ± 3.7

^Δ^ *F* = 28.9, *p* < 0.01; ^※^ *F* = 79.2, *p* < 0.01.

### 2.3. Prediction of Target Genes of miRNA-548ah and GO or Pathway Analysis

**Figure 2:**
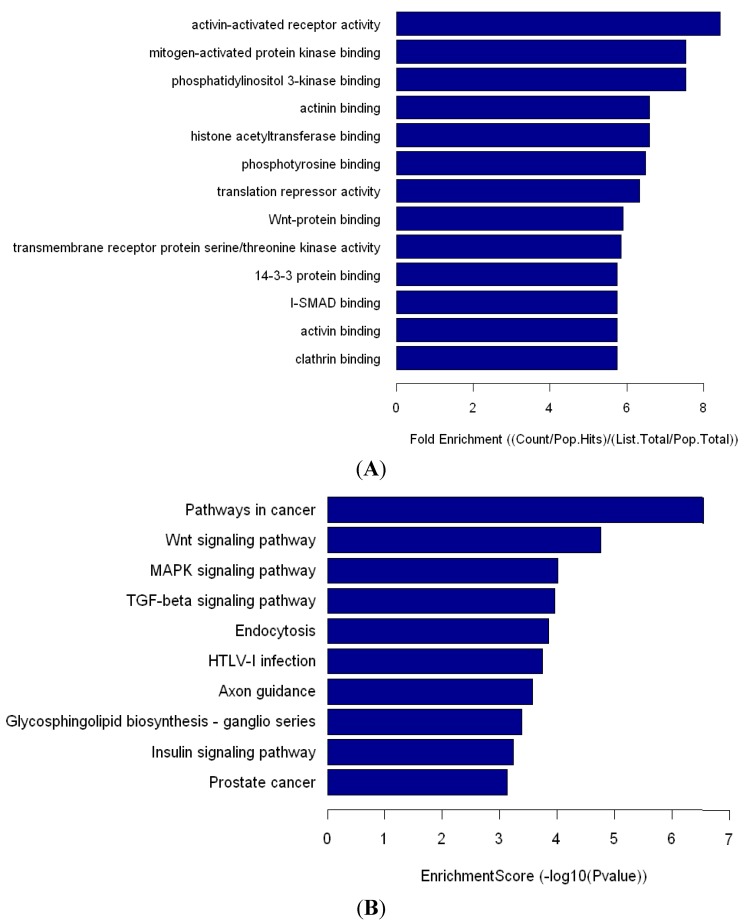
Molecular function of Go analysis of miR-548ah target gene (**A**); Pathway analysis of miR-548ah target gene (**B**).

The intersection of miRNA-548ah target genes is analyzed by TargetScan, miRDB and miRadna (Lewis BP, Cambridge, MA, USA; Wang XW, St. Louis, MO, USA; Enright AJ, New York, NY, USA; respectively). The number of miRNA-548ah target genes is 195. GO analysis showed that several molecular functions were significantly featured in numerous protein binding activities (Figure 2A). Pathway analysis showed that miRNA-548ah may participate in numerous signaling pathways, such as the Wnt signaling pathway, the MAPK signaling pathway, the TGF-β signaling pathway, and many other pathways (Figure 2B).

### 2.4. IFN-γR1 Is Direct Target Gene of miR-548ah-5p

**Figure 3:**
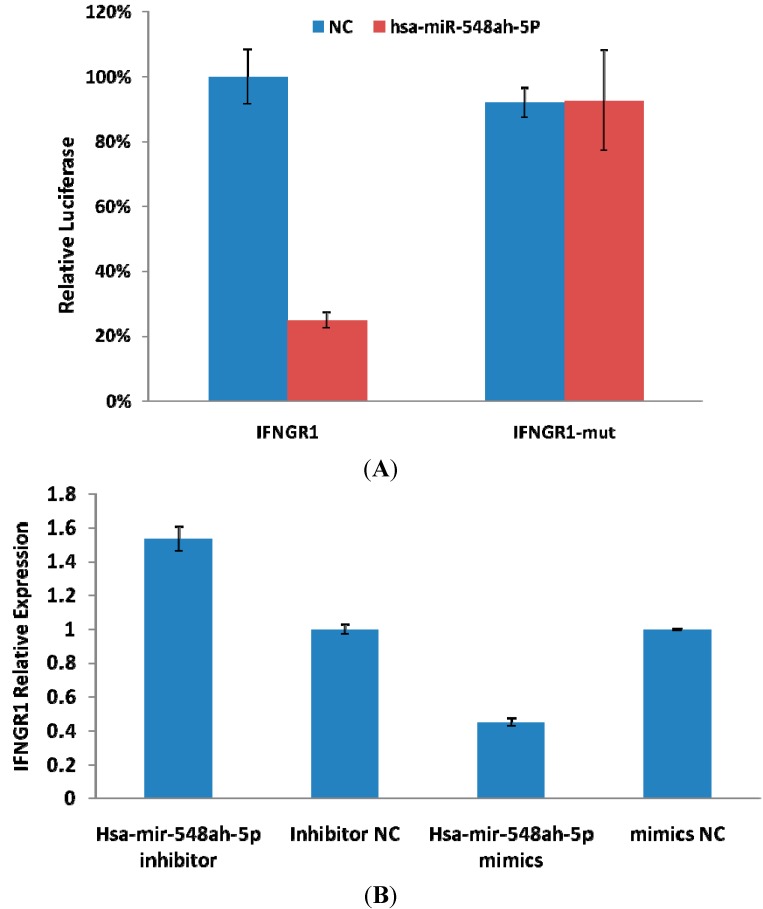
Suppressed luciferase activity of wild type 3'-UTR of IFN-γR1 by miR-548ah mimic (**A**); The expression of IFN-γR1 mRNA in THP-1 cells regulated by miR-548ah mimics and inhibitor (**B**).

**Figure 4:**
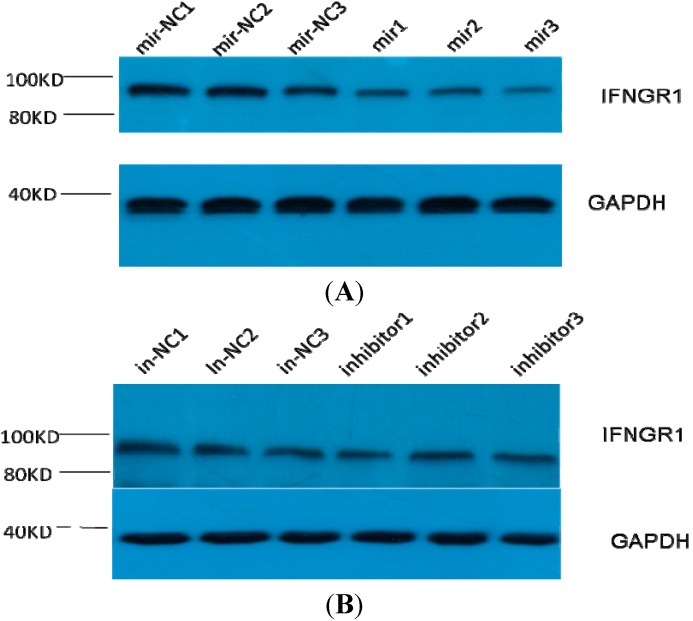
Expression of endogenous IFN-γR1 in THP-1 cells by miR-548ah mimics (**A**) and inhibitors (**B**). Glyceraldehyde-3-phosphate dehydrogenase (GAPDH) was used as an internal control. Legend: mir-NC 1 to 3 and In-NC 1 to 3 are normal control of miR-548ah-5p; mir1 to 3 are miR-548ah-5p mimic; inhibitor 1 to 3 are miR-548ah-5p inhibitors.

IFN-γR1 was predicted as a potential target gene of the miR-548ah-5p. The 3'-UTR of IFN-γR1 contained two complementary sites for the seed region of miR-548ah-5p. To validate whether IFN-γR1 is a direct target of miR-548ah-5p, the 3'-UTR fragment of IFNγR1 containing wild-type or mutant miR-548ah-5p binding sequence was cloned downstream of the firefly luciferase reporter gene in psiCHECK-2 plasmids. Among the 293 T cells cotransfected with the reporter plasmids and miR-548ah-5p mimic or NC duplex, the luciferase activity of the reporter that contained wild-type 3'-UTR was significantly suppressed by the miR-548ah-5p mimic; however the luciferase activity of the mutant reporter was unaffected by transfection (Figure 3A). Furthermore, transfection of the miR-548ah-5p mimic or inhibitor decreased or increased IFN-γR1 expression in THP-1 cells at mRNA levels (Figure 3B). Levels of IFN-γR1 protein expression in THP-1 cells decreased by the transfection of miR-548ah-5p mimics (0.54 ± 0.03 *vs.* 0.31 ± 0.02; *t* = 6.29, *p* < 0.01) (Figure 4A). No significant effect on the levels of IFN-γR1 protein expression in THP-1 cells were found by the transfection of miR-548ah-5p inhibitors (0.54 ± 0.02 *vs.* 0.55 ± 0.04; *t* = 0.22, *p >* 0.05) (Figure 4B).

### 2.5. Correlation of miR-548ah with ALT, HBV DNA and IFN-γR1 mRNA of Patients with CHB

**Figure 5:**
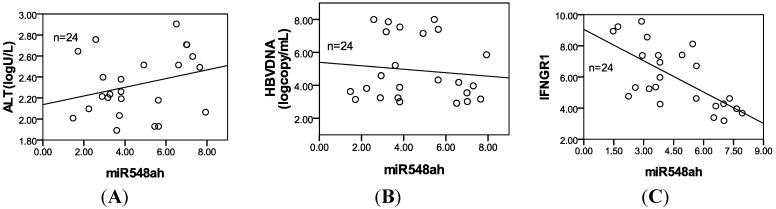
Scatter diagram of correlation between miR548ah (Δ*C*_t_) and three indicators of CHB patients. (**A**) ALT (*r* = 0.28, *p* > 0.05); (**B**) HBVDNA (*r* = −0.11, *p* > 0.05); (**C**) IFN-γR1 (Δ*C*_t_) (*r* = −0.67, *p* < 0.01).

The 24 CHB patients had an ALT level of 2.3 ± 0.3 (logarithm values) and an HBV DNA level of 4.9 ± 1.9 (logarithm values). No significant correlation was found between miR-548ah, ALT, and HBV DNA(*r* = 0.28, −0.11, *p* > 0.05) (Figure 5A,B). The mRNA levels of IFN-γR1 in IA phase of CHB were 5.9 ± 2.0 (Δ*C*_t_). Significant negative correlation was found between the levels of miR-548ah and mRNA levels of IFN-γR1 (*r* = −0.67, *p* < 0.01) (Figure 5C).

## 3. Discussion

Some studies have reported that the interaction between certain miRNAs and HBV has an important function in the pathogenesis of chronic HBV infection [18,19]. Results from recent research has shown that the miRNA expression of PBMC were associated with the severity of HBV-induced liver disease and therapeutic outcome of IFN-α therapy in CHB patients [20,21]. Winther’s reports first indicated the existence of a relationship between abundance of circulating miRNAs and immunological stages in the natural course of CHB. Certain miRNAs may contribute to the establishment and maintenance of CHB in children [22]. In this study, the expression profiles of miRNA molecules in the IT and IA phases of CHB were detected using miRNA microarray. The results showed significantly different expression profiles of miRNA molecules among the IT and IA phases of CHB and healthy controls. Significant up- and downregulation of miRNA molecules were observed in the IA phase of CHB, which may indicate partial recovery of immune function. The overall miRNA expressions were downregulated in the IT phase of CHB. This may be attributed to inhibition of miRNA expression and dysfunction of organism-specific immune response resulting from high HBV levels. Five upregulated miRNAs, especially hsa-miR-548ah-5p and hsa-miR-4804-3p (>5 times), were found between the IA and IT phases of CHB and verified through RT-PCR. Thus, our results first revealed that the abnormal expression of miRNAs in PBMC are closely correlated with various immune states of CHB patients. The abnormal expression of certain miRNA molecules may have important functions in the damage of the immune homeostatic mechanisms of chronic HBV infection.

Human miR-548 is a large gene family that includes 69 members. Based on the predicted target mRNAs of hsa-miR-548, functional enrichment analyses show that the miR-548 gene family functions in multiple biological processes, including various human diseases [23]. Li *et al*. [24] found that hsa-miR-548 family members are highly associated with the impaired IFN signaling of CHB. Similarly, the expression level of hsa-miR-548ah-5p is significantly increased in the IA phase of CHB. miRNA-548 may down-regulate host antiviral response via direct targeting of IFN-λ1 [25]. Pathway analysis showed that miR-548ah may participate in numerous signaling pathways, such as the Wnt signaling pathway, the MAPK signaling pathway, and the TGF-β signaling pathway. The significantly upregulated expression of hsa-miR-548ah-5p may be related to changes in immune functions from the IT phase to the IA phase of CHB. However, no correlation was found between the hsa-miR-548ah, ALT and HBV DNA. Analysis of the reasons may be related to the following factors: (a) The number of samples are small; (b) Different means of detection; (c) Affection of other abnormal expression of miRNA molecules, *etc*. Hsa-miR-4804 is one of the newly discovered microRNAs and is located in Chromosome 5. The function of hsa-miR-4804 and its role in the pathogenesis of chronic hepatitis B is unclear at present and needs further study.

Interferon (IFN)-γ is an important cytokine that increases immune system activity. It is secreted by active Th1 cells and by NK cells. Tasi *et al*., study have showed that the activation of Th1 immunity accompanied by enhancement of CTL activity during therapy is a common immune mechanism for successfully treating hepatitis B [26]. Functions of IFN-γ are completed upon binding to the receptors of IFN-γ. The receptors of IFN-γ include two subunits: IFN-γR1 and IFN-γR2. IFN-γR1 was predicted as a potential target gene of miR-548ah-5p. The mRNA and protein levels of endogenous IFN-γR1 could be inhibited by the mimics of miR-548ah-5p in the THP-1 cell lines. However, levels of endogenous IFN-γR1 protein did not increase from the transfection of miR-548ah-5p inhibitors into THP-1 cells, which may be related to the lower expression of endogenous miR-548ah-5p. Luciferase analysis showed that miR-548ah-5p could regulate the expression of human IFN-γR1 by directly targeting IFN-γR1 mRNA. Significant negative correlation was found between the miR-548ah and mRNA levels of IFN-γR1 in CHB patients. These results suggest that IFN-γR1, as a target gene of miR-548ah-5p, influences the molecular function of IFN-γ through inhibition of IFN-γR1 expression. This inhibition of miR-548ah-5p will reduce the function of IFN-γ and seems to be contradicted with the immune activation of CHB patients. However, considering the control function of miRNA is the “tuners” mechanism, rather than “switchers” mechanism, this way might be related with regulation of hepatic inflammation activity degree in CHB patients. Furthermore, the interaction between other miRNAs(miR-122, *etc*.) and IFN should be taken into account in this course [21,27].

In summary, miRNA expression profiles are significantly different in various PBMCs and may be closely associated with immune activation of chronic HBV infection. miR-548ah, by targeting IFN-γR1, may represent a mechanism that can facilitate viral pathogenesis and help determine novel therapeutic molecular targets.

## 4. Methodology

### 4.1. Subjects

Blood samples were collected from 24 patients with chronic HBV infection as well as 9 healthy people, who formed the control group, at the Taizhou People’s Hospital from 2011 to 2012. Among the 24 chronic HBV infection patients, 12 had CHB in the IT phase and 12 had CHB in the IA phase. The baseline data of the three groups are shown in Table 2. The quantitative PCR validation group consisted of 47 patients with chronic HBV infection admitted at the Taizhou People’s Hospital from 2012 to 2013. 23 patients had CHB in the IT phase, while 24 had CHB in the IA phase. The control group consisted of 24 healthy patients. The baseline data of the three groups are shown in Table 3. Written informed consent was obtained from all subjects. The experimental protocol was approved by the ethical commission of Taizhou People’s Hospital, and diagnostic criteria were based on the 2010 Chronic Hepatitis B Prevention Guide of China [28]. All patients were negative for antibodies against hepatitis A, C, D, and E viruses as well as human immunodeficiency virus. All patients with a history and clinical features of drug-induced liver injury, alcoholic hepatitis, and steatohepatitis as well as those treated with nucleotide or nucleotide-analog antiviral or immunomodulatory drugs in the previous six months were excluded from this study.

**Table 2:** Baseline data of group detected by microarray.

	IT of CHB	IA of CHB	HC
**Sex (m/f)**	4/8	3/9	2/7
**Age (year)**	29 ± 7	33 ± 8	30 ± 6
**ALT (U/L)**	25.8 ± 7.1	216.5 ± 273.5	24.1 ± 6.2
**HBV DNA (Log^10^copies/mL)**	6.8 ± 1.4	5.7 ± 1.5	-

**Table 3:** Baseline data of group detected by RT-qPCR.

	IT of CHB	IA of CHB	HC
**Sex (m/f)**	14/9	19/5	15/9
**Age (year)**	31 ± 7	38 ± 10	29 ± 9
**ALT (U/L)**	23 ± 8	265 ± 188	16 ± 10
**HBV DNA (Log^10^copies/mL)**	7.1 ± 1.3	4.9 ± 1.9	-

### 4.2. PBMC Separation and miRNA Microarrays

#### 4.2.1. PBMC Separation

PBMCs were separated by Ficoll-Hypaque gradient centrifugation. 5 × 10^6^ PBMCs were collected, added 1 mL TRIzol (Invitrogen, Carlsbad, CA, USA) and frozen at −80 °C until examined. The PBMCs of three subjects from the same group were pooled and four or three pools were analyzed in the microarray groups.

#### 4.2.2. RNA Extraction and Labeling

Total RNA was isolated using TRIzol (Invitrogen, Carlsbad, CA, USA) and miRNeasy mini kits (QIAGEN, Duesseldorf, Germany) according to the manufacturer’s instructions. All RNA species, including miRNA molecules, were efficiently recovered. RNA was determined and measured using a nanodrop spectrophotometer (ND-1000, Nanodrop Technologies, Wilmington, DE, USA). RNA integrity was determined by gel electrophoresis. A miRCURY™ Hy3™/Hy5™ Power labeling kit (Exiqon, Vedbaek, Denmark) was used for miRNA labeling according to the manufacturer’s instructions.

#### 4.2.3. Array Hybridization

Hy3™-labeled samples were hybridized by a miRCURY™ LNA array (v.18.0) kit (Exiqon, Vedbaek, Denmark) according to the manufacturer’s instructions. Briefly, a mixture of 25 μL of Hy3™-labeled samples and 25 μL of hybridization buffer was denatured for 2 min at 95 °C and incubated on ice for 2 min. The mixture was then hybridized to the microarrays for 16–20 h at 56 °C in a 12-bay hybridization system (Hybridization System, Nimblegen Systems, Inc., Madison, WI, USA). This system provides an active mixing action and a constant incubation temperature to improve the hybridization uniformity and enhance signals. After hybridization, the slides were washed several times using a wash buffer (Exiqon, Vedbaek, Denmark) and then dried by centrifugation for 5 min at 400 rpm. The slides were scanned using an Axon GenePix 4000B microarray scanner (Axon Instruments, Foster City, CA, USA).

#### 4.2.4. Data Analysis

The scanned images were imported into GenePix Pro 6.0 software (Axon Instruments, Foster City, CA, USA) for grid alignment and data extraction. The replicated miRNA molecules were averaged, and miRNA molecules with intensities of ≥50 in the samples were selected to calculate the normalization factor. The expressed data were then normalized by median normalization. Significantly and differentially expressed miRNA molecules were identified by volcano plot filtering. Hierarchical clustering was performed using MEV software (v4.6, TIGR, Rockville, MD, USA). There was a significant upward or downward trend when the standard value of experimental group 1.5 times higher than or 0.67 times lower than that of normal control group (*p* < 0.05).

### 4.3. Detection of miRNA Molecules and IFNγR1mRNA Using Real-Time Quantitative PCR

#### 4.3.1. Synthesis of cDNA

Briefly, 2 μL of 10× reverse transcriptase (RT) buffer, 1.2 μL of 1 μM of RT-specific primers, 2 μL of total RNA, and 0.2 μL of 200 U/μL Moloney murine leukemia virus RT were mixed in water and diluted to a total volume of 20 μL. The reaction for RT-PCR amplification was performed as follows: 42 °C for 30 min and 85 °C for 10 min.

#### 4.3.2. Quantitative Real-Time PCR

The primers were synthesized by Shanghai Invitrogen Biotechnology Co., Ltd. The primer sequences of U6, hsa-miR-548ah-5p and hsa-miR-4804-3p are F: 5'ATTGGAACGATACAGAGAAGATT3', R: 5'GGAACGCTTCACGAATTTG3'; GSP: 5'GAAGTTCCATCGAAAAGTGATTG3', R: 5'TATGCTTGTTCTCGTCTCTGTGTC3'; GSP: 5'GCCCTGCTTAACCTTGCCCT3', R: 5'TGCAGGGTCCGAGGT3'; respectively. Briefly, 10 μL of 2× Master Mix, 0.08 μL of 20 μM PCR specific primer F and 0.08 μL of 20 μM PCR specific primer R, were mixed with water to a total volume of 18 μL. The mixture was then added to each hole of a 384-PCR plate corresponding followed by addition of 2 μL of cDNA. The mixture was carefully glued using parafilm and briefly centrifuged. The 384-PCR plate was placed on an RT-PCR instrument prior to the PCR process. U6 small nuclear RNA molecule was used as internal controls. The reactions for U6, hsa-miR-548ah-5p and hsa-miR-4804-3p were performed as follows: 95 °C for 3 min, followed by 40 cycles of 95 °C for 12 s and 62 °C for 40 s. The primer sequences of IFN-γR1 (human NM_000416) are F: 5'AATAGCAGTATAAAAGGTTCTC3', R: 5'CTTACCACAGAGATCAAGGAC3'. Briefly, 0.4 μL ROX Reverse Dye (50×), 10 µL SYBR premix ex taq, 0.4 μL of 10 μM PCR specific primer F, 0.4 μL of 10 μM PCR specific primer R and 2 μL cDNA, were mixed with water to a total volume of 20 μL. Beta-Actin was used as internal controls. The reaction for IFN-γR1 and Beta-Actin were performed as follows: 95 °C for 30 s, followed by 40 cycles of 95 °C for 5 s and 62 °C for 34 s.

#### 4.3.3. Data Analysis

Data were calculated using the 2^−ΔΔ*C*t^ method. Δ*C*_t_ = miR548 (4804) *C*_t_-*U*6 *C*_t_. The higher the Δ*C*_t_ value, the lower the expression obtained.

### 4.4. Detection of the Main Clinical Indicators

Quantitative detection of HBV DNA was performed using an ABI7300-type quantitative PCR instrument (Applied Biosystems, Carlsbad, CA, USA). HBV markers were detected using an enzyme-linked immunosorbent assay (Beijing YuanPingHao Biological Company Limited, Beijing, China). Biochemical indicators were detected using an automatic biochemical analyzer (Hitachi Ltd., Tokyo, Japan).

### 4.5. Prediction of miRNA-548ah Target Gene

Possible target genes of miRNA-548ah were predicted by Target Scan [29], miRDB [30], and miRadna [31]. The intersection of these three databases was taken. The accessed time was on 16 January 2014.

### 4.6. GO and KEGG Analysis of miRNA-548ah Possible Target Genes

miRNA targets were subjected to gene ontology (GO) [32] analysis to uncover the miRNA-gene regulatory network on the basis of biological processes and molecular functions. Enrichment provided a measure of the significance of the function. Pathway analysis was used to determine the significant pathway of the differential genes according to the Kyoto encyclopedia of genes and genomes (KEGG) [33]. Fisher’s exact and *X*^2^ test were used to select the significant pathway. The false discovery rate (FDR) was calculated to correct the *p* value. *p* < 0.05 was considered to statistically significant difference. The accessed time was on 20 January 2014.

### 4.7. Culture and Transfection of THP-1 Cell Lines

THP1 is a human monocytic cell line derived from an acute monocytic leukemia patient. THP-1 cell lines (obtained from Shanghai Institute for Biological Science, Shanghai, China) were cultured in RPMI 1640 with 10% FBS, 100 U/mL penicillin, and 100 μg/mL streptomycin at 37 °C with 5% carbon dioxide. The miR-548ah mimic, miR-548ah inhibitor, and unrelated sequence positive and negative controls were purchased from GenePharma (Shanghai GenePharma Co., Ltd., Shanghai, China). Cells were transfected using Lipofectamine Transfection Reagent (Life Technology, Carlsbad, CA, USA) and were harvested 48 h later. The expression levels of miR-548ah and IFNγR1mRNA were detected in THP-1 cells using qRT-PCR. The protein expression levels of IFNγR1 were measured in the cells through Western blotting.

### 4.8. Western Blotting

Protein extracts from the THP-1 cells were prepared using a modified radioimmunoprecipitation buffer with 0.5% sodium dodecyl sulfate in the presence of proteinase inhibitor cocktail. Protein weighing 50 µg was electrophoresed in 10% sodium dodecyl sulfate-polyacrylamide gel electrophoresis minigels and transferred onto polyacrylamide fluoride membranes. After blocking with 5% bovine serum albumin, the membranes were incubated with mouse anti-IFNγR1 antibody (Santa Cruz Biotechnology, Santa Cruz, CA, USA) or rabbit anti-glyceraldehyde-3-phosphate dehydrogenase (GAPDH) antibody (Beyotime, Shanghai, China) at 4 °C overnight, followed by incubation with horseradish peroxidase-conjugated goat anti-mouse antibody (Santa Cruz, CA, USA) or goat anti-rabbit antibody (Beyotime, Nantong, China) for 1 h at room temperature. Signals were developed using a chemoluminescent substrate (Amersham Biosciences Corp., Piscataway, NJ, USA) and visualized through X-ray films. The gray value of each specific band on the images was digitized using Image J analysis software (NIH, Bethesda, MD, USA). Protein gray values were divided by the internal reference GAPDH to correct errors. Results of a sample represent the relative protein content.

### 4.9. Construction and Transfection of Luciferase Reporter Plasmids

The 3'-untranslated region (UTR) fragment of IFNγR1 (138–359 nt, Genbank accession number NM_000416), which contains two putative miR-548ah binding sequences (162–168 and 326–332 nt), was amplified with the primers. The PCR products were cloned into the firefly luciferase reporter vector psiCHECK-2 (Promega Corporation, Madison, WI, USA) called psiCHECK-IFNγR1. Plasmids carrying the mutated sequence in the complementary sites for the seed regions of IFNγR1 were generated based on psiCHECK-IFNγR1 plasmids through site-specific mutagenesis called psiCHECK-IFNγR1-mut. Transfection was carried out using Lipofectamine transfection reagent (Invitrogen, Carlsbad, CA, USA) following the manufacturer’s protocol. In brief, 5 × 10^4^ 293T cells in a 48-cell plate were transfected with the indicated miRNA mimic (Shanghai GenePharma, Shanghai, China) or plasmid DNA and collected 48 h after transfection for assay. Luciferase activity was measured using a dual-luciferase reporter assay kit (Promega Corporation, Madison, WI, USA) and recorded using a multi-plate reader (GloMax, Promega, Madison, WI, USA).

### 4.10. Statistical Analysis

Data were expressed as mean ± standard deviation. The *t*-test was used for comparison between two groups, while one-way ANOVA and SNK-q tests were used for multiple comparisons. The The *X*^2^ test was used for count data. Pearson correlation was used between variables. Data analysis was performed using SPSS17.0 statistical software (SPSS, Inc., Chicago, IL, USA). *p* < 0.05 was considered to indicate statistically significant difference.

## Abbreviations

HBV: hepatitis B virus
miRNA: MicroRNA
CHB: chronic hepatitis B
IT: immune tolerance
IA: immune activation
PCR: polymerase chain reaction
